# Increased risk for other cancers in individuals with Ewing sarcoma and their relatives

**DOI:** 10.1002/cam4.2575

**Published:** 2019-10-31

**Authors:** Diana Abbott, Schuyler O'Brien, James M. Farnham, Erin L. Young, Jeffrey Yap, Kevin Jones, Stephen L. Lessnick, R. Lor Randall, Joshua D. Schiffman, Lisa A. Cannon‐Albright

**Affiliations:** ^1^ Genetic Epidemiology Department of Internal Medicine University of Utah School of Medicine Salt Lake City UT USA; ^2^ Huntsman Cancer Institute University of Utah Salt Lake City UT USA; ^3^ Department of Orthopedic Surgery University of Utah Salt Lake City UT USA; ^4^ Department of Radiology University of Utah Salt Lake City UT USA; ^5^ Center for Childhood Cancer and Blood Diseases at Nationwide Children's Hospital Division of Pediatric Hematology/Oncology/Blood and Marrow Transplant The Ohio State University College of Medicine Columbus OH USA; ^6^ Department of Orthopedic Surgery UC Davis Sacramento CA USA; ^7^ Division of Pediatric Hematology/Oncology Department of Pediatrics University of Utah Salt Lake City UT USA; ^8^ George E. Wahlen Department of Veterans Affairs Medical Center Salt Lake City UT USA

**Keywords:** cancer, Ewing sarcoma, relative, relative risk, UPDB

## Abstract

**Background:**

There are few reports of the association of other cancers with Ewing sarcoma in patients and their relatives. We use a resource combining statewide genealogy and cancer reporting to provide unbiased risks.

**Methods:**

Using a combined genealogy of 2.3 million Utah individuals and the Utah Cancer Registry (UCR), relative risks (RRs) for cancers of other sites were estimated in 143 Ewing sarcoma patients using a Cox proportional hazards model with matched controls; however, risks in relatives were estimated using internal cohort‐specific cancer rates in first‐, second‐, and third‐degree relatives.

**Results:**

Cancers of three sites (breast, brain, complex genotype/karyotype sarcoma) were observed in excess in Ewing sarcoma patients. No Ewing sarcoma patients were identified among first‐, second‐, or third‐degree relatives of Ewing sarcoma patients. Significantly increased risk for brain, lung/bronchus, female genital, and prostate cancer was observed in first‐degree relatives. Significantly increased risks were observed in second‐degree relatives for breast cancer, nonmelanoma eye cancer, malignant peripheral nerve sheath cancer, non‐Hodgkin lymphoma, and translocation sarcomas. Significantly increased risks for stomach cancer, prostate cancer, and acute lymphocytic leukemia were observed in third‐degree relatives.

**Conclusions:**

This analysis of risk for cancer among Ewing sarcoma patients and their relatives indicates evidence for some increased cancer predisposition in this population which can be used to individualize consideration of potential treatment of patients and screening of patients and relatives.

## Graphical abstract

We used a unique resource linking genealogy from the mid 1800s to a statewide cancer registry from the mid 1960s to explore the association of cancers of other sites among Ewings sarcoma patients and their relatives. While this rare cancer was not observed to cluster in close relatives, multiple other cancer sites were observed among patients and their relatives and this data could inform the recommended cancer screening for this population.

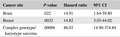

## 1 INTRODUCTION

Ewing sarcoma is a rare tumor occurring most often in the bones or soft tissues of children and young adults. Ewing sarcoma accounts for ~1.5% of all childhood cancers and, although uncommon, is still considered the second most common type of bone tumor in children. One common feature of Ewing sarcoma tumors is the presence of a translocation involving EWSR1 and a member of the E26 transformation‐specific (ETS) transcription factor family, most commonly FLI1 (about 85% of patients).^1^, ^2^, ^3^, ^4^ Although the genetic epidemiology of Ewing sarcoma is not entirely clear, race remains a known significant risk factor for Ewing sarcoma, with incidence in Caucasians nine times greater than in Africans (0.155 vs 0.017 per 10^5^ individuals).^5^ Further supporting an element of genetic risk, despite an overall incidence of just one case per million, there are several reports of Ewing sarcoma observed in siblings.^6^, ^7^, ^8^, ^9^ Based on this evidence, genetic risk loci have been sought through multiple genome‐wide association study efforts, but there is no consensus as to what degree these risk candidates contribute to Ewing sarcoma development.^10^, ^11^, ^12^ Other studies have investigated germline mutations in known cancer predisposition genes, finding a minority (11%) of patients to have pathogenic, or probably pathogenic, variants in *TP53*, *RET*, and *PMS2* 13; family history does not predict the presence of a syndrome in most patients. A pathogenic role for these mutations in Ewing sarcoma has not been demonstrated.^14^ Specific environmental exposures have not been recognized as risk factors, although an association with the occupation of farming in parents has been suggested,^15^, ^16^, ^17^, ^18^ and hernias in childhood have been associated with increased risk^19^, ^20^, ^21^; this association with hernias is unclear but may indicate common neuroectodermal pathways contributing to risk.

There have been few studies reporting the risk of other cancers in Ewing sarcoma patients or their relatives. Ji and Hemminki^22^ reported significantly increased risk for Ewing sarcoma (SIR = 16.5; 1.60, 60.8) when a sibling was diagnosed with Ewing sarcoma; in addition, a significantly increased risk of Ewing sarcoma was observed for parental kidney cancer (SIR = 5.6 (1.5, 14.6). Novakovic^23^ reported no overall increased risk of cancer among 4628 first‐ or second‐degree relatives of 256 Ewing sarcoma patients; however, several specific tumor types were observed in significant excess, including stomach cancer (RR = 2.0; 1.4, 2.8); melanoma (RR = 1.9; 1.2, 2.8); brain tumor (RR = 1.9; 1.1, 3.0); and bone cancer (RR = 4.2; 1.7, 8.6). Risks of these cancers were higher among maternal than paternal relatives, but not significantly. Given Ewing sarcoma's rarity as well as the inaccuracies often associated with familial cancer questionnaires, unbiased identification of overrepresented cancers among relatives of Ewing sarcoma patients is challenging. Here we utilize a unique Utah population‐based resource to expand the characterization of risk for cancers of other sites in Ewing sarcoma patients and in their relatives.

## 2 MATERIALS AND METHODS

### 2.1 Utah population database

The Utah Population Database (UPDB) includes a computerized genealogy of the original Utah pioneers and their descendants to modern day.^24^, ^25^ The genealogical data have been linked to a statewide cancer registry from 1966, allowing identification of all relationships among cancer patients. The genealogical data today extend to 16 generations. To ensure similarity with respect to membership in the population and record linking quality for all comparisons, analysis of data within the UPDB is limited here to individuals with at least three generations of genealogy data who link to original Utah founders. In the UPDB data analyzed here, there are 2.3 million individuals with at least three generations of genealogy, and these individuals are linked to approximately 123 000 Utah cancer records.

### 2.2 Utah Ewing Sarcoma patients

The Utah Cancer Registry (UCR) was formed in 1966; in 1973, it became one of the now 11 registries that make up the National Cancer Institute's Surveillance, Epidemiology, and End Results (SEER) program. Data are collected using a standardized set of definitions and codes. All cancers reported to the UCR are manually consolidated and edited by certified tumor registrars. Data only for all independent primary cancers diagnosed or treated in Utah are collected and stored in the UCR, including primary site, histology, behavior, age at diagnosis, stage, grade, survival, and treatment, among others; data on cancer recurrence are not reported or stored. Ewing sarcoma patients who linked to at least three generations of genealogy in the UPDB were identified from UCR records based on the International Classification of Diseases for Oncology (ICD‐O) Revision 3 with histology 9260 (n = 130), or 9364 (n = 13).

### 2.3 Estimation of risk for cancer

To estimate relative risk (RR) among relatives of Ewing sarcoma cancer patients for cancer, by site, the disease rate for each cancer site in the UPDB was estimated independently. The definitions for cancers analyzed are seen in Table S1 and include only primary site, histology, and behavior coding. Because cancer rates differ by sex, may change over time, and are generally lower in Utah, cohort‐specific cancer rates were estimated from individuals in the UPDB who have at least three generations of genealogy data. These individuals were assigned to a specific cohort that was determined by sex, year of birth (5 year cohorts), and birth state (Utah or not). Cohort‐specific rates for each cancer site were estimated by dividing the number of cancer patients in each cohort by the total number of individuals in each cohort. The RR for cancer of any site in a group of individuals was estimated as the ratio of the observed number of cancer patients for the site of interest to the expected number of cancer patients among the group of individuals. The observed number of cancer patients among the group of individuals was summed over all cohorts, the expected number of patients was calculated by multiplying the number of individuals in each cohort by the cohort‐specific rate of the cancer of interest, and summing over all cohorts. One‐sided *P*‐values indicating the probability of observing an excess number of a specific cancer were calculated under the assumption that the number of observed cancers follows a Poisson random variable with a mean equal to the expected number of cancers. This same RR estimation procedure was used for each cancer site, and for each set of relatives (first‐, second‐, and third‐degree).

Because individuals with Ewing sarcoma have a decreased life expectancy, their years at risk for developing cancer is lower than that of the general population; this decreased time at risk must be accounted for by modifying the estimation of risk for cancer. Therefore, in contrast to the estimation of cancer risk in relatives, to calculate RRs for cancer for the Ewing sarcoma patients, each Ewing sarcoma case was matched, without replacement, to 100 cohort‐matched controls with appropriate genealogy data. A Cox proportional hazards model in R using the *coxphf* package was performed, and distinct survival functions were fit, by cancer site, for Ewing sarcoma patients, and for controls. For each cancer site, the time to event was defined as the number of years (in 1‐year increments) it took for an individual to be diagnosed with the cancer of interest. Censoring occurred when individuals died without being diagnosed with a specific cancer. For each cancer site, the dependent variable “time to diagnosis” was modeled as being determined by an individual's Ewing sarcoma case status and UPDB cohort covariate profile. After fitting a Cox proportional hazards model, a hazard rate for the cancer of interest was determined for patients and for controls. The hazard ratio (HR) was calculated as the ratio of the case rate to the control rate, and signifies an Ewing sarcoma case's increased risk per 1‐year time period for developing a specific cancer. Risks for 38 different cancer sites for which there were at least 200 patients recorded in the UCR (Table S1) were estimated for Ewing sarcoma patients and for their first‐, second‐, and third‐degree relatives.

## 3 RESULTS

In the UCR, 143 individuals diagnosed with Ewing sarcoma were identified who had at least three generations of genealogy; 90 (63%) patients were male (average age of diagnosis 20.9 years ± 13.6; median age = 32 years) and 53 (37%) were female (average age of diagnosis 17.6 years ± 11.3; median age = 10 years); 96 patients (67%) were no longer living and had an average age of death of 26.4 years ± 14.8. The remaining 47 patients had an average current age of 33.7 years ± 14.3. The 143 Ewing sarcoma patients had a total of 744 first‐degree relatives, 2228 second‐degree relatives, and 6552 third‐degree relatives.

Cancers of four different primary cancer sites other than Ewing sarcoma (breast, brain, complex genotype/karyotype sarcoma, and unknown site) were observed in at least one Ewing sarcoma case. For those Ewing sarcoma patients where data were available for age at diagnosis for both cancers, 50% of the other cancers occurred at an average of 18 years after the diagnosis of Ewing sarcoma, and 50% of the other cancers occurred at an average of 2 years before the diagnosis of Ewing sarcoma. Table 1 summarizes HR estimates for the cancer sites that were observed to be significantly elevated in Ewing sarcoma patients. Due to the small sample sizes and to ensure confidentiality, no data on the sample sizes or characteristics of the other cancers are provided.

**Table 1:** Estimated hazard ratios for cancers observed in significant excess in the 143 individuals diagnosed with Ewing sarcoma

Cancer site	*P*‐value	Hazard ratio	95% CI
Brain	.022	14.91	1.64‐59.80
Breast	.0032	14.82	3.03‐44.02
Complex genotype/karyotype sarcoma	.00006	86.03	14.90‐374.84

Note

Number of events <5 so data not shown.

Within the UPDB, Ewing sarcoma patients had a total of 744 first‐degree, 2228 second‐degree, and 6,552 third‐degree relatives who also had at least three generations of genealogy data. RR estimates for cancers that were observed in significant excess among first‐, second‐, and third‐degree relatives of the 143 Ewing Sarcoma patients are summarized in Table 2; for any cancer site observed in excess in any degree of relationship, results are shown for all degrees of relationship. No Ewing sarcoma patients were identified among first‐, second‐, or third‐degree relatives of Ewing sarcoma patients. The data indicate significantly increased risk for lung/bronchus, female genital, and prostate cancer in first‐degree relatives. Significantly increased risks were observed in second‐degree relatives for breast cancer, nonmelanoma eye cancer, malignant peripheral nerve sheath cancer, non‐Hodgkin lymphoma, and translocation sarcomas. Significantly increased risk for stomach cancer, prostate cancer, and acute lymphocytic leukemia was observed in third‐degree relatives. Small sample sizes may have limited this analysis, but some consistency can be observed. Non‐Hodgkin lymphoma and prostate cancer were observed in excess in all degrees of relationship, although the excess was not always statistically significant. Similarly, acute lymphoblastic leukemia and malignant peripheral nerve sheath cancers were observed in excess in two of the three degrees of relationship, although not always with statistical significance. Conversely, the significant excesses observed for lung/bronchus and female genital cancers in first‐degree relatives were not observed in excess in the other degrees of relationship, suggesting the likelihood of common environmental effects or multiple testing issues.

**Table 2:** Estimated RRs for cancers of other sites observed in significant excess among first‐, second‐, and third‐degree relatives of individuals diagnosed with Ewing sarcoma

Cancer site	Obs/Exp	*P*‐value	RR	One‐sided 95% CI
Ewing sarcoma first‐degree relatives (n = 744)				
Breast	6/8.0	.32	0.75	0.00‐1.48
Nonmelanoma eye	0/0	.94	—	
Non‐Hodgkin lymphoma	**	.43	1.78	0.09‐9.90
Lung/bronchus	7/2.8	.023	2.53	1.19‐5.21
Female genital	**	.045	5.97	1.06‐21.55
Stomach	**	.44	1.73	0.09‐9.66
Prostate	16/8.7	.017	1.84	1.15‐2.99
Translocation sarcoma	0/0.2	.86	—	
Malignant peripheral nerve sheath	**	.05	18.52	0.95‐103.19
Acute lymphocytic leukemia	**	.28	3.04	0.16‐16.95
Brain	**	.09	2.92	0.60‐8.52
Ewing sarcoma Second‐degree relatives (n = 2228)				
Breast	44/29.1	.006	1.51	1.16‐2.03
Nonmelanoma eye	**	.019	9.47	1.68‐34.20
Non‐Hodgkin lymphoma	16/7.8	.01	2.06	1.29‐3.35
Lung/bronchus	9/11.9	.26	0.76	0.00‐1.32
Female genital	**	.66	0.82	0.00‐3.91
Stomach	**	.21	0.34	0.00‐1.60
Prostate	42/34.7	.13	1.21	0.92‐1.63
Translocation sarcoma	**	.039	6.61	1.16‐23.51
Malignant peripheral nerve sheath	**	.009	14.59	2.59‐52.71
Acute lymphocytic leukemia	0/0.8	.45	—	
Brain	6/2.92	.08	2.06	0.75‐4.47
Ewing sarcoma third‐degree relatives (n = 6552)				
Breast	72/74.4	.42	0.97	0.00‐1.18
Nonmelanoma eye	**	.42	1.83	0.09‐10.21
Non‐Hodgkin lymphoma	37/33.3	.29	1.11	0.83‐1.53
Lung/bronchus	29/32.1	.33	0.9	0.00‐1.23
Female genital	**	.38	0.62	0.00‐1.96
Stomach	16/9.3	.029	1.71	1.08‐2.78
Prostate	117/95.3	.017	1.23	1.05‐1.47
Translocation sarcoma	0/0.8	.47	—	
Malignant peripheral nerve sheath	0/0.4	.69	—	
Acute lymphocytic leukemia	7/2.0	.005	**3.46**	1.62‐7.11
Brain		12/7.2	1.66	0.86‐2.91

** <5 patients observed; data not shown.

## 4 DISCUSSION

We previously reviewed the evidence of a heritable predisposition for Ewing sarcoma and concluded that more data were needed in the field.^26^ This analysis of risk of cancer for other sites among Ewing sarcoma patients and their relatives in a unique genealogical resource indicates evidence for some increased cancer predisposition in this population. The observation of coaggregation of cancer of different sites in Ewing sarcoma patients and in their relatives could provide evidence for shared genetic etiology, and thereby enhance knowledge of cancer predisposition. Although sample sizes for this and previous studies remain relatively small, overlap exists with previously reported findings with respect to increased risk for stomach cancer, and several novel observations have been made.

Significantly elevated HRs for cancers of several other sites were observed for Ewing sarcoma patients (Table 1). Secondary malignant neoplasms are known to be associated with Ewing sarcoma; however, the degree to which therapy contributes to this risk is difficult to determine.^27^, ^28^, ^29^ Breast cancer has previously been reported as one such secondary malignant neoplasm seen in excess among Ewing sarcoma patients.^30^, ^31^ While some of this risk is likely attributed to radiation fields in the chest area, increased risk in the absence of radiation to the chest area has also been demonstrated. A recent report shows that the *EWS‐FLI1* transcription causes R‐loops and blocks *BRCA1* repair in Ewing sarcoma, perhaps explaining the observed increased risk of breast cancer.^32^ Our study identified excess breast cancer risk in Ewing sarcoma patients (Table 1) and in their second‐degree relatives (Table 2), further supporting the hypothesis of an associated inherited risk for breast cancer. It is possible that increased screening in Ewing sarcoma patients and their relatives might partially explain observed excesses.

Literature noting brain cancer as a secondary malignant neoplasm in Ewing sarcoma is less complete. Similar to breast cancer, brain cancer was seen in excess in not only Ewing sarcoma patients (Table 1), but in their first‐degree relatives as well (Table 2); and was observed to be elevated, but not statistically significantly so, in second‐ and third‐degree relatives. This suggests a potential inherited predisposition for both breast and brain cancer, as increased risk in relatives of Ewing sarcoma patients cannot be attributed to secondary malignancies related to treatment of Ewing sarcoma. These results are based on small sample sizes, but the lower limit of the confidence intervals for the significantly elevated HRs (ranging from 1.77 to 23.06) as well as the excess risk observed in relatives indicate that some special attention to risk for these other cancers occurring among Ewing sarcoma patients and their relatives might be warranted.

Results for risk of cancers of other sites in both the close and distant relatives of Ewing sarcoma patients identified some other cancer sites of interest. Prostate cancers were observed in excess among first‐ and third‐degree relatives. This seems especially relevant to Ewing sarcoma as prostate cancers frequently harbor ETS gene fusions.^33^, ^34^ Whether a common risk factor may contribute to such aberrant ETS gene fusions remains unknown, but this observation provides one potential link between Ewing sarcoma and prostate cancer etiology. Again, sample sizes are small, and additional studies could clarify these results.

Differences from other investigations on the risk of cancer in Ewing sarcoma patients and their relatives include the use of genealogic data to define relationships and the use of an NCI SEER cancer registry for cancer diagnosis, in contrast to other studies' reliance on self‐reported family history and cancer status. It must be noted that after an appropriate correction for multiple testing for the 37 cancer sites examined in three degrees of relationship, none of the observed excesses remain statistically significant.

Limitations of this study include the small number of Ewing sarcoma patients with adequate genealogy data (n = 143). Complete Utah cancer data are only available from 1973, providing a small window of diagnoses that does not allow complete consideration of relatives born in different generations. Cancers occurring outside the state of Utah are also censored. Missing or incorrect genealogy data are possible; genealogy data does not always represent biological relationships. The Utah population analyzed is almost entirely of Northern European ancestry, so findings should not be extrapolated further. Strengths of this study include that all UCR cancer patients have pathologic confirmation and are carefully vetted, and while some diagnoses and relationships may have been censored, the results are unbiased.

This study suggests that other cancers may be observed in excess in Ewing sarcoma patients and their relatives. Some of the cancers identified in excess here are recognized to be associated with the known cancer genes reported to be observed in 11% of Ewing sarcoma patients (*TP53*, *RET*, and *PMS2*); a search for pathogenic variants in some of the patients and relatives identified in this study could clarify this. Identifying and validating these cancers that are consistently seen in excess in Ewing sarcoma patients and their relatives could aid in narrowing down genetic risk if a common and overlapping risk mechanism is responsible. Furthermore, if validated, this information could be used to reduce the risk of increased secondary malignancies in Ewing sarcoma, and also to design early tumor surveillance strategies. Similar to many other Ewing sarcoma studies, despite the use of the UPDB, our investigation was still limited in statistical power by sample size. Follow‐up studies with a focus on cancer history in Ewing sarcoma probands and their relatives would reinforce these findings and give more weight to clinical action based on the excess tumors identified in patients and families with Ewing sarcoma.

## CONFLICT OF INTEREST

The authors have no conflict of interest with the information presented.

## AUTHOR CONTRIBUTIONS

Diana Abbott: formal analysis, methodology, software, supervision, writing‐original draft, writing‐review and editing; Schuyler O'Brien: data curation, resources, writing‐review and editing; James Farnham: data curation, formal analysis, methodology, software; writing original draft, writing‐review and editing; Erin L. Young: resources, writing‐review and editing; Jeffrey Yap: resources, writing‐review and editing; Kevin Jones: resources, writing‐review and editing; Stephen L. Lessnick: resources, writing‐review and editing; R. Lor Randall: resources, writing‐review and editing; Joshua D. Schiffman: funding acquisition, resources, supervision, writing‐review and editing; Lisa A. Cannon‐Albright: conceptualization, funding acquisition, methodology, project administration, software, supervision, writing original draft and review and editing.
