# Supplementary material for: Increased risk for other cancers in individuals with Ewing sarcoma and their relatives
Source: Cancer Med. 2019 Oct 31;8(18):7924–30. doi: 10.1002/cam4.2575 (PMC6912049; doi:10.1002/cam4.2575)
Supplement: Supplementary file 1 [file CAM4-8-7924-s001.docx]

**Supplemental Table 1. Cancer site definitions by ICD-Oncology Revision 3 site, morphology and behavior**

| **Cancer Type** | **Site** | **Morphology** | **Behavior** |
| --- | --- | --- | --- |
| Lip | 0-9 | 8000-9589 | 2-9 |
| Tongue | 19-29 | 8000-9589 | 2-9 |
| Salivary | 79-89 | 8000-9589 | 2-9 |
| Pharynx | 90-119, 129-39, 140-48 | 8000-9589 | 2-9 |
| Esophagus | 150-159 | 8000-9589 | 2-9 |
| Stomach | 160-169 | 8000-9589 | 2-9 |
| Small intestine | 170-179 | 8000-9589 | 2-9 |
| Colon | 180, 182-189, 260 | 8000-8239, 8250-8719 | 3-9 |
| Rectum | 199, 209 | 8000-8239, 8250-8719 | 3-9 |
| Anus | 210-218 | 8000-9589 | 3-9 |
| Liver/hepatic | 220, 221 | 8000-9589 | 2-9 |
| Gallbladder | 239 | 8000-9589 | 2-9 |
| Pancreas | 250-259 | 8000-1, 8010, 8140-1, 8480-1, 8500 | 2-9 |
| Larynx | 320-329 | 8000-9589 | 2-9 |
| Lung/bronchus | 340-349 | 8000-9589 | 2-9 |
| Bone | 400-419 | 8000-9589 | 2-9 |
| Melanoma | 0-809 | 8720-8790 | 2-9 |
| Breast | 500-509 | 8000-9589 | 2-9 |
| Cervix | 530-539 | 8000-9589 | 2-9 |
| Endometrial | 540-549, 559 | 8010, 8050, 8140, 8210, 8260, 8310, 8323, 8380, 8382, 8441, 8460-1, 8480-81, 8560, 8570 | 2-9 |
| Ovary | 569 | 8000-9589 | 2-9 |
| Female Genital | 510-519, 529, 570-79 | 8000-9589 | 2-9 |
| Prostate | 619 | 8000-9589 | 3-9 |
| Testis | 620-629 | 8000-9589 | 2-9 |
| Bladder | 670-679 | 8000-9589 | 2-9 |
| Kidney | 649, 659 | 8000-9589 | 2-9 |
| Brain/CNS | 710-729 | 8000-9529, 9540-9589 | 2-9 |
| Thyroid | 739 | 8000-9589 | 2-9 |
| Complex genotype/  Karyotype sarcoma | 0-809 | 8804, 8810, 8811, 8825, 8830, 8850, 8854, 8890, 8901, 8910, 8912, 8990, 9120, 9180-9185, 9187, 9192-9195, 9220-9221, 9240, 9242, 9243, 9261, 9370-9372 | 3 |
| Translocation  sarcoma | 0-809 | 8806, 8814, 8833, 8852-3, 8920, 9040-1,9043-4, 9231, 9260, 9363, 9364, 9365, 9581 | 3 |
| Multiple myeloma | 0-809 | 9732 | 2-9 |
| Chronic lymphocytic leukemia | 0-809 | 8823 | 2-9 |
| Acute lymphocytic leukemia | 0-809 | 9821, 9828, 9831-7 | 2-9 |
| Chronic myelocytic leukemia | 0-809 | 9863, 9875, 9876 | 2-9 |
| Acute myelocytic leukemia | 0-809 | 9840, 9861, 9866-7, 9871-4, 9891, 9895-7, 9920 | 2-9 |
| Non-Hodgkins lymphoma | 0-809 | 9530, 9590-1, 9596, 9598, 9670-1, 9673, 9675, 9678-80, 9684, 9687, 9689-91, 9695, 9698-702, 9705, 9708-9, 9714, 9716-9, 9727-29, 9755, 9764, 9827 | 2-9 |
| Hodgkins lymphoma | 0-809 | 9650-5, 9659, 9661-7, 9945-6, 9948 | 2-9 |
| Oropharynx | 19, 24, 51-52, 90-91, 98-104, 108-9 | 8000-9589 | 2-9 |
